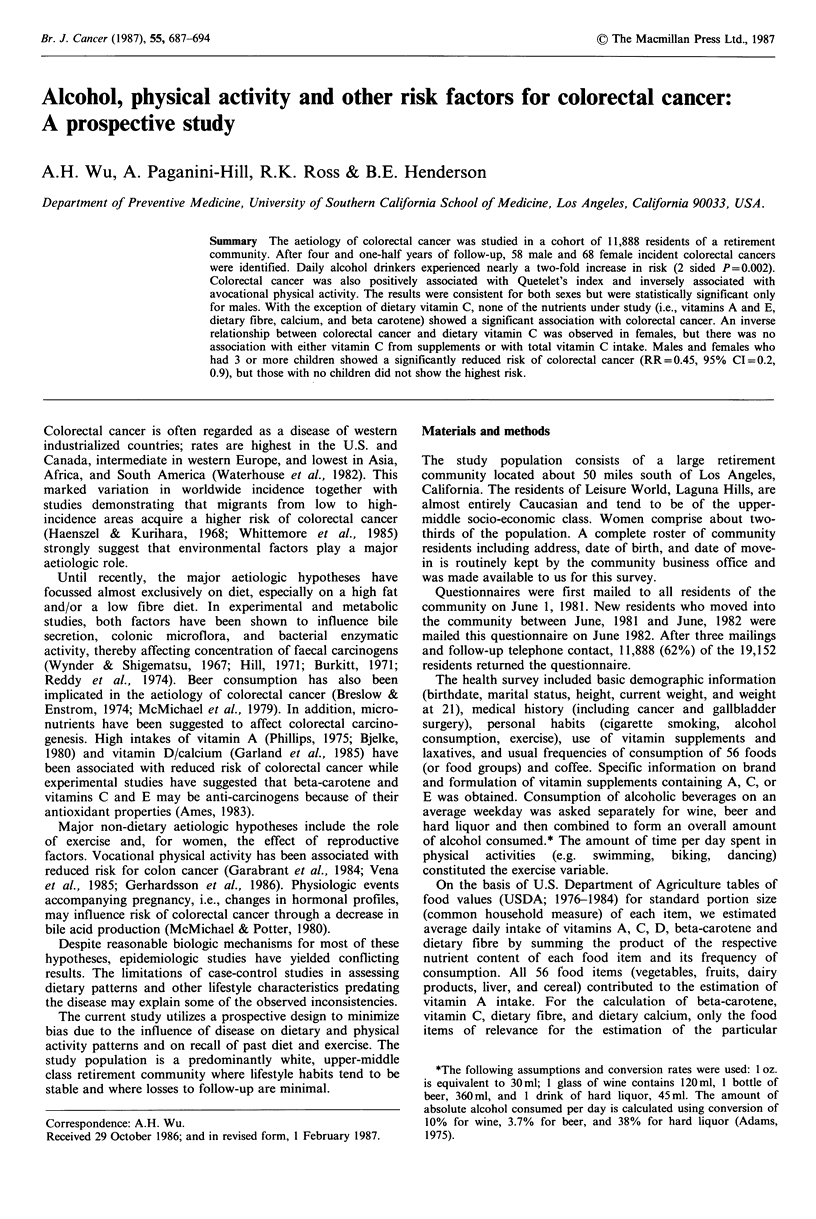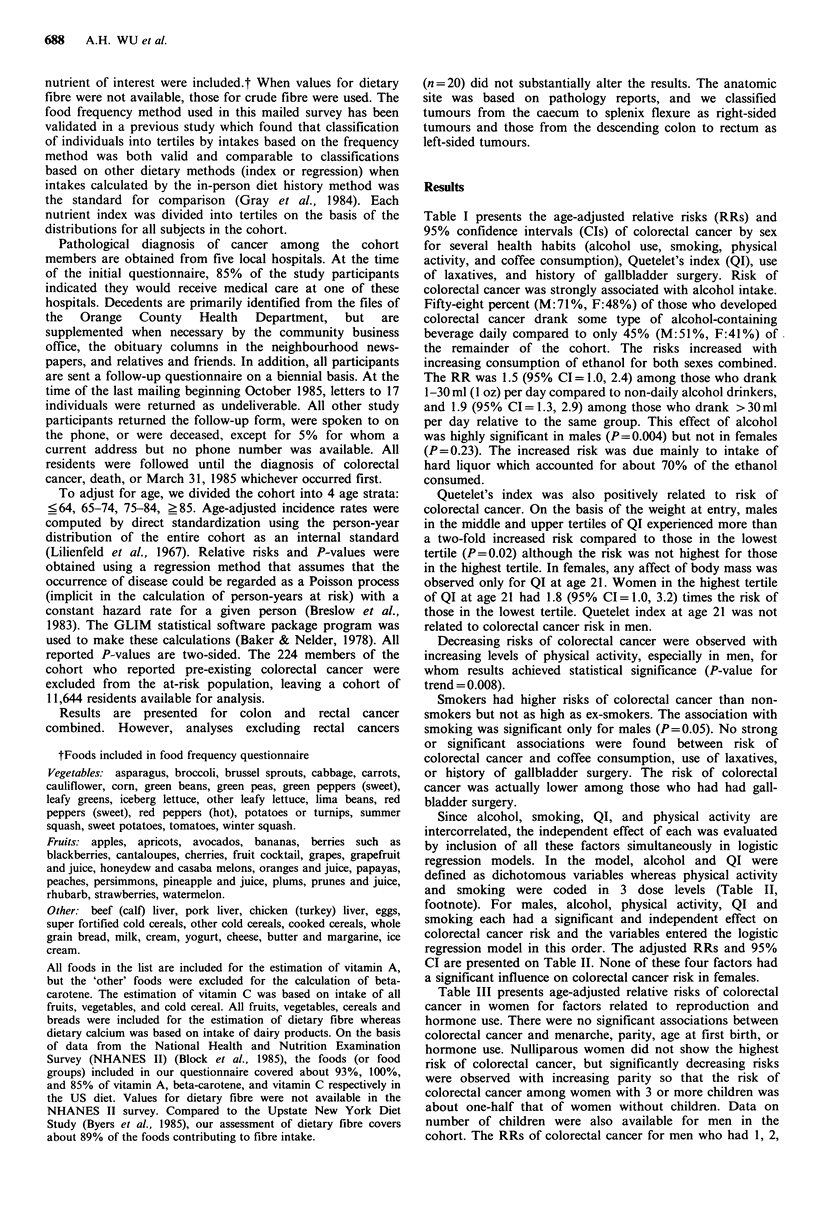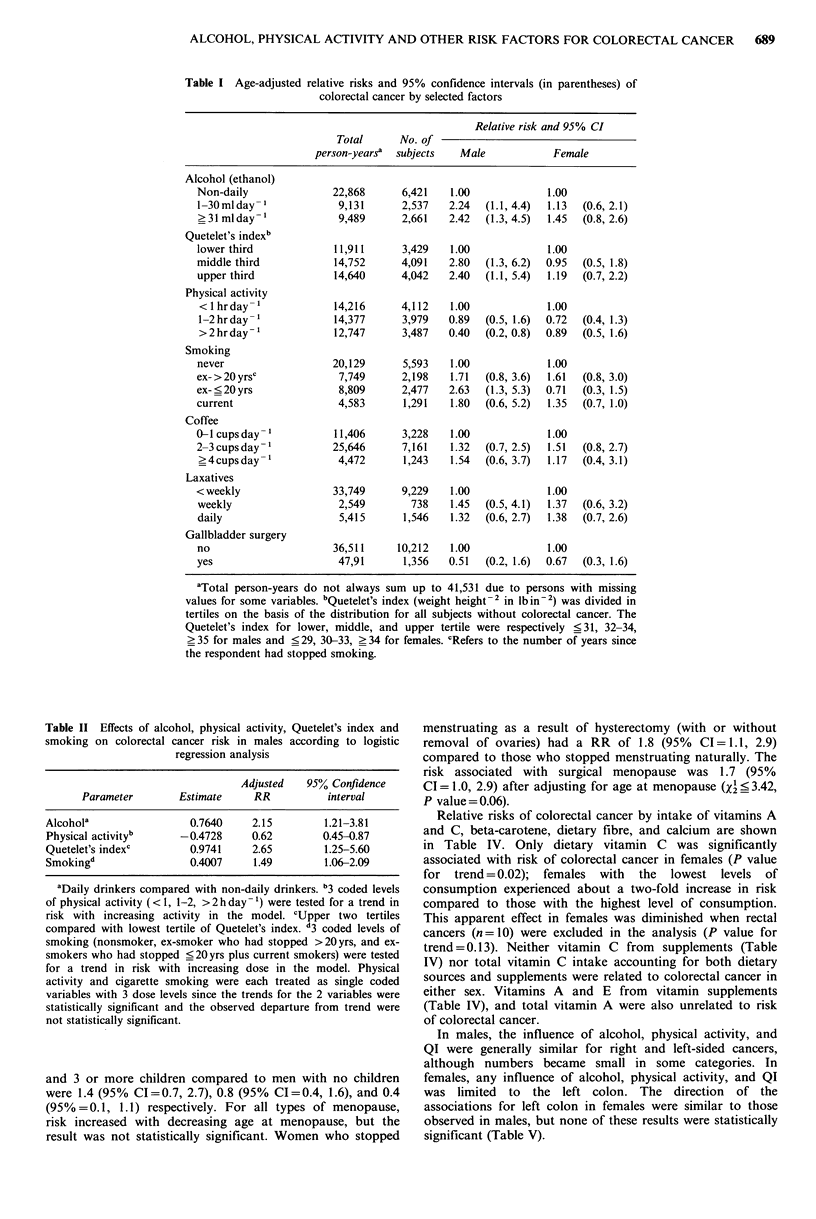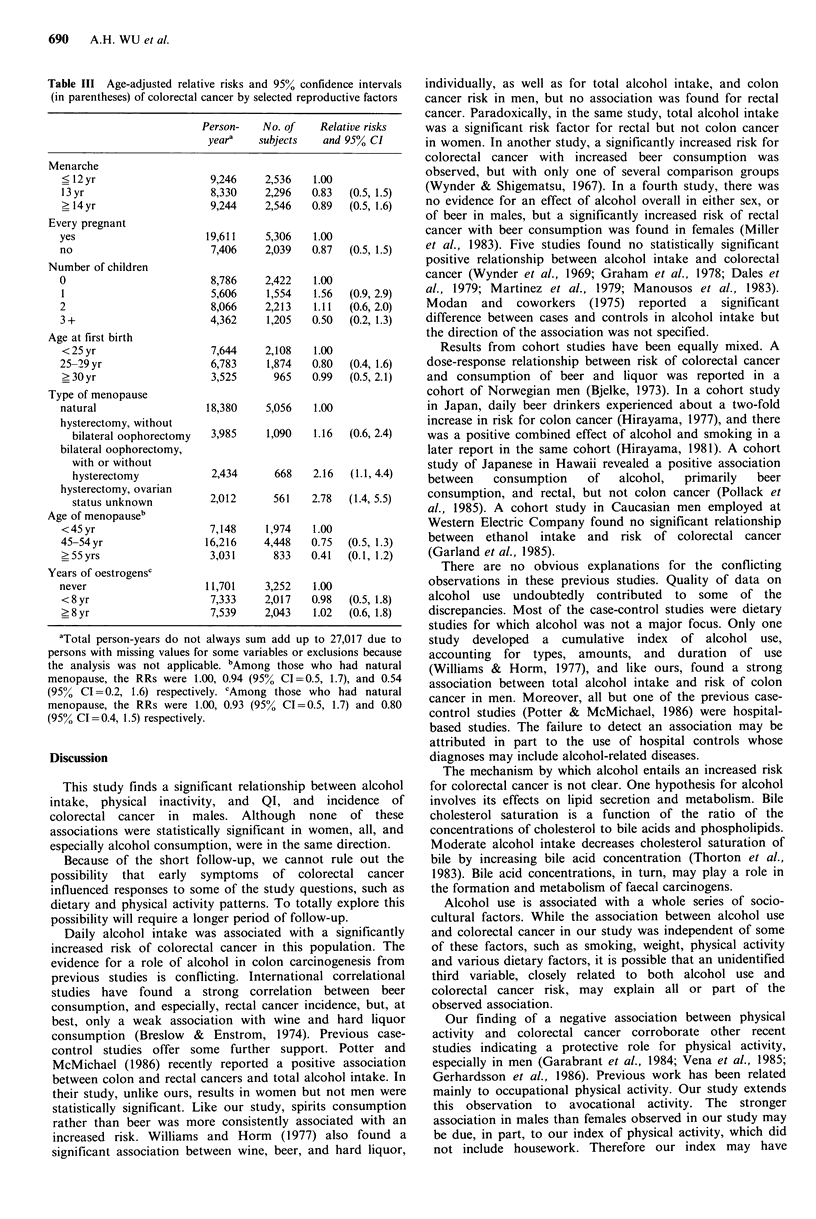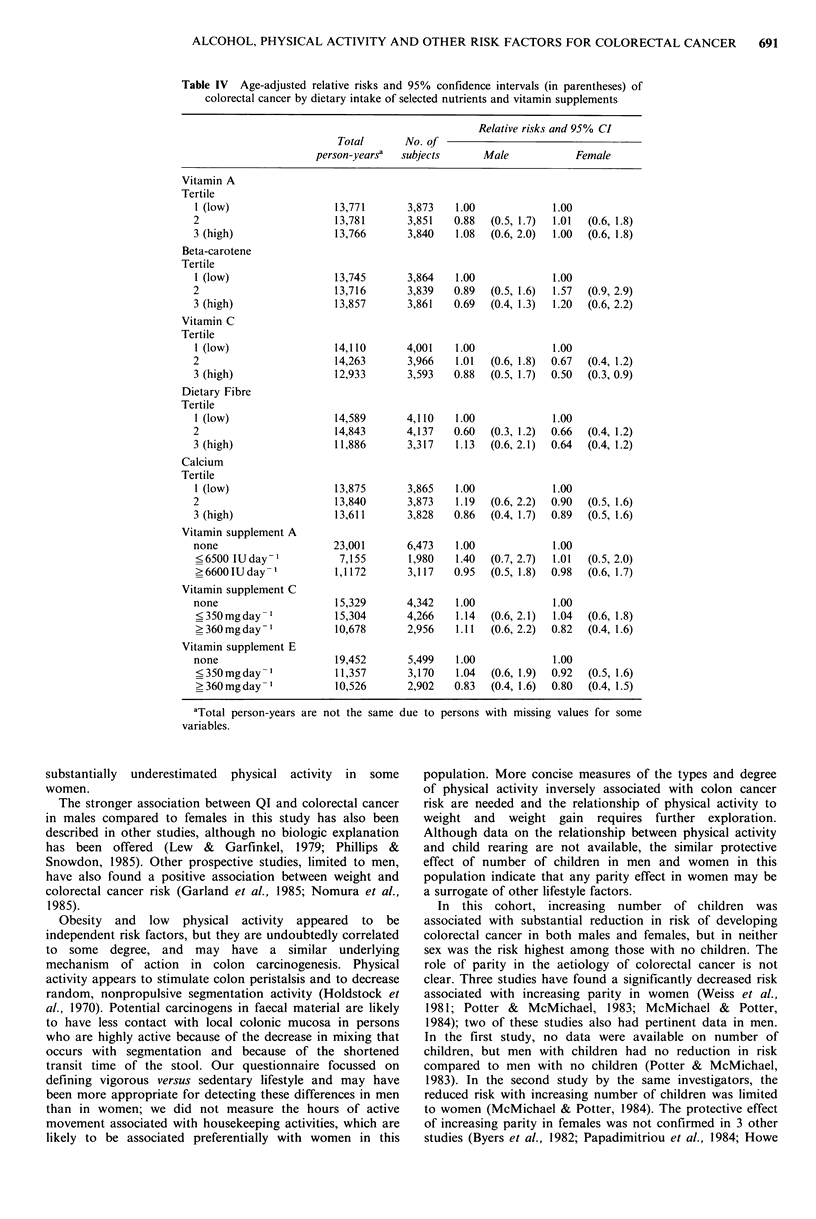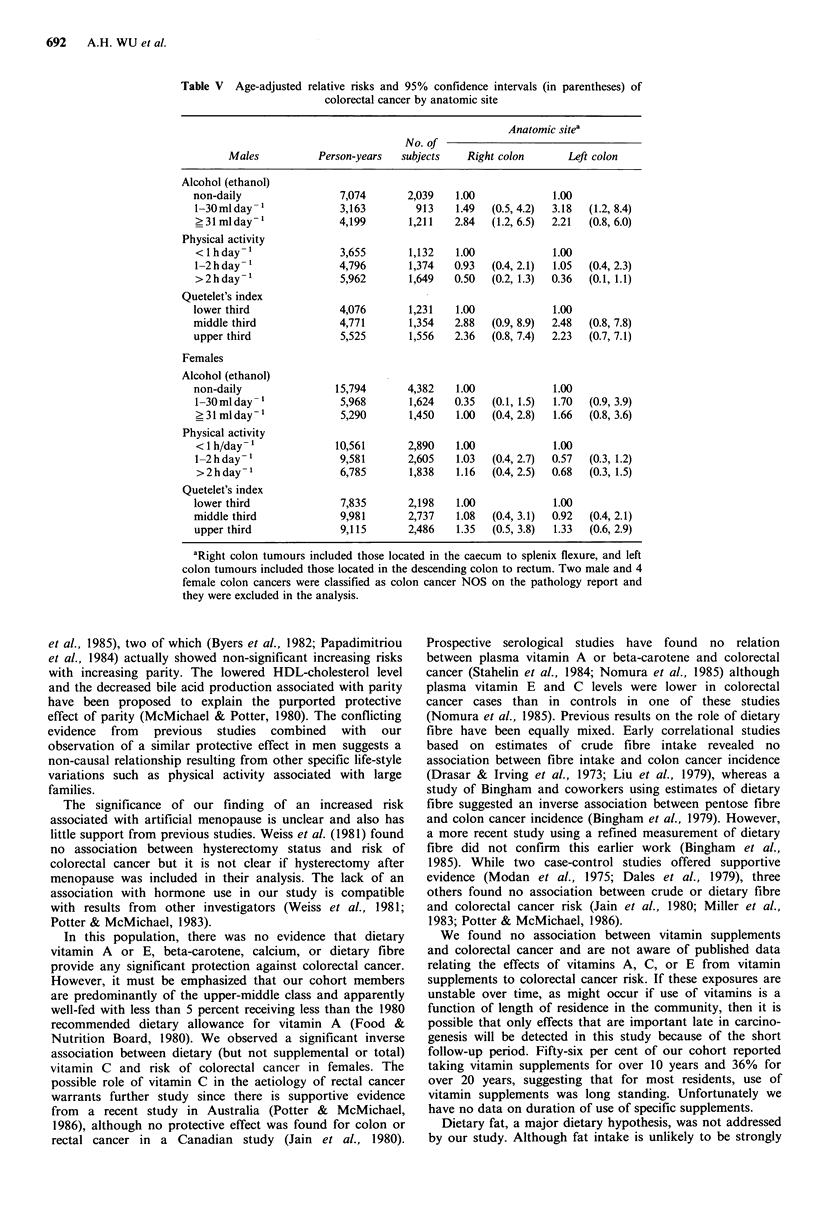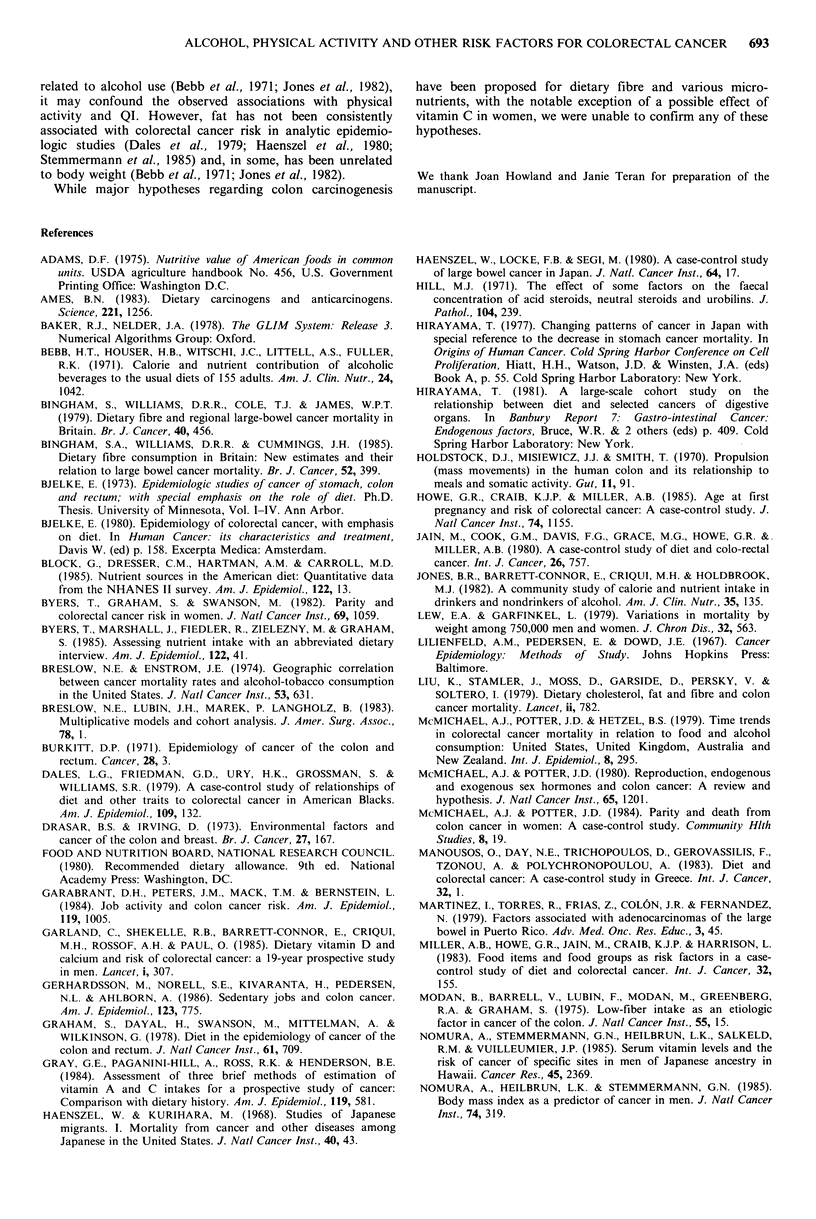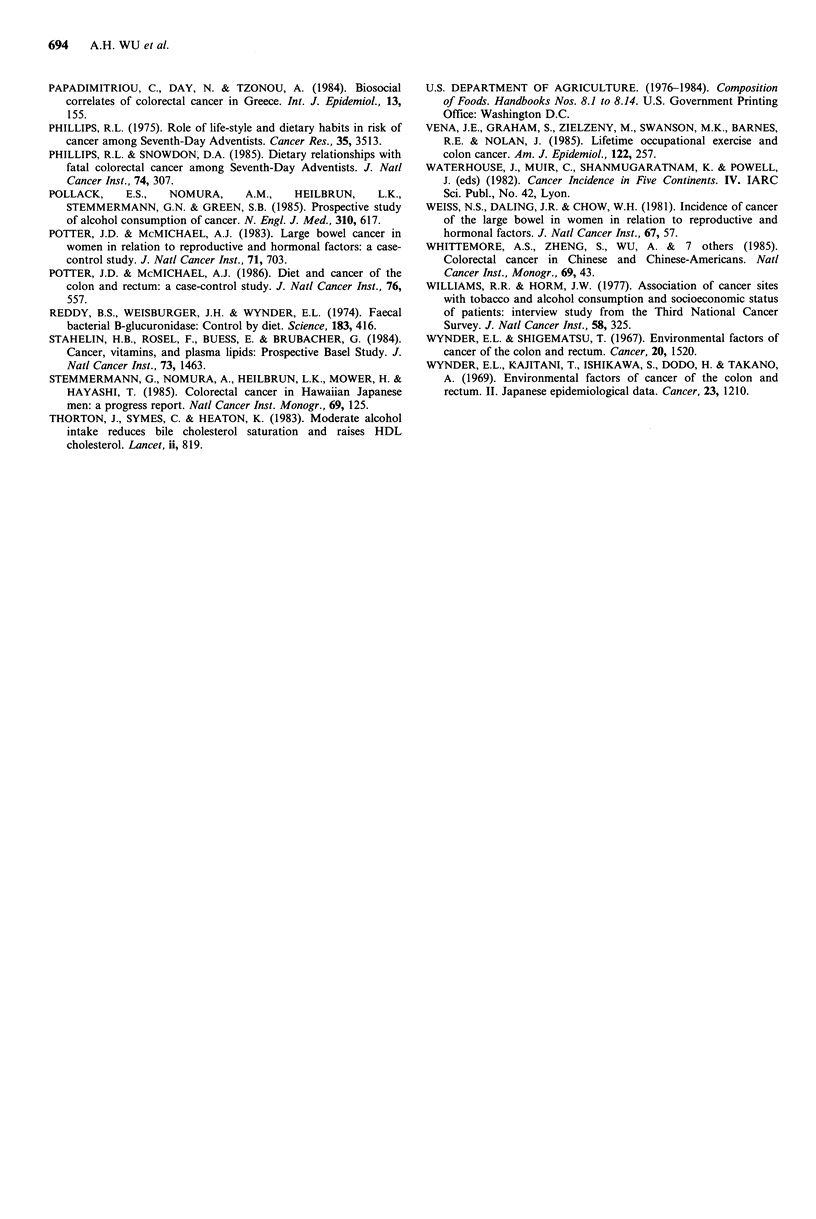# Alcohol, physical activity and other risk factors for colorectal cancer: a prospective study.

**DOI:** 10.1038/bjc.1987.140

**Published:** 1987-06

**Authors:** A. H. Wu, A. Paganini-Hill, R. K. Ross, B. E. Henderson

## Abstract

The aetiology of colorectal cancer was studied in a cohort of 11,888 residents of a retirement community. After four and one-half years of follow-up, 58 male and 68 female incident colorectal cancers were identified. Daily alcohol drinkers experienced nearly a two-fold increase in risk (2 sided P = 0.002). Colorectal cancer was also positively associated with Quetelet's index and inversely associated with avocational physical activity. The results were consistent for both sexes but were statistically significant only for males. With the exception of dietary vitamin C, none of the nutrients under study (i.e., vitamins A and E, dietary fibre, calcium, and beta carotene) showed a significant association with colorectal cancer. An inverse relationship between colorectal cancer and dietary vitamin C was observed in females, but there was no association with either vitamin C from supplements or with total vitamin C intake. Males and females who had 3 or more children showed a significantly reduced risk of colorectal cancer (RR = 0.45, 95% CI = 0.2, 0.9), but those with no children did not show the highest risk.


*Br. J. Cancer* (1987), **55**, 687–694
© The Macmillan Press Ltd., 1987

### Alcohol, physical activity and other risk factors for colorectal cancer: A prospective study

A.H. Wu, A. Paganini-Hill, R.K. Ross & B.E. Henderson

*Department of Preventive Medicine, University of Southern California School of Medicine, Los Angeles, California 90033, USA.*

**Summary** The aetiology of colorectal cancer was studied in a cohort of 11,888 residents of a retirement community. After four and one-half years of follow-up, 58 male and 68 female incident colorectal cancers were identified. Daily alcohol drinkers experienced nearly a two-fold increase in risk (2 sided $P=0.002$). Colorectal cancer was also positively associated with Quetelet's index and inversely associated with avocational physical activity. The results were consistent for both sexes but were statistically significant only for males. With the exception of dietary vitamin C, none of the nutrients under study (i.e., vitamins A and E, dietary fibre, calcium, and beta carotene) showed a significant association with colorectal cancer. An inverse relationship between colorectal cancer and dietary vitamin C was observed in females, but there was no association with either vitamin C from supplements or with total vitamin C intake. Males and females who had 3 or more children showed a significantly reduced risk of colorectal cancer (RR=0.45, 95% CI=0.2, 0.9), but those with no children did not show the highest risk.

Colorectal cancer is often regarded as a disease of western industrialized countries; rates are highest in the U.S. and Canada, intermediate in western Europe, and lowest in Asia, Africa, and South America (Waterhouse *et al.*, 1982). This marked variation in worldwide incidence together with studies demonstrating that migrants from low to high-incidence areas acquire a higher risk of colorectal cancer (Haenszel & Kurihara, 1968; Whittemore *et al.*, 1985) strongly suggest that environmental factors play a major aetiologic role.

Until recently, the major aetiologic hypotheses have focussed almost exclusively on diet, especially on a high fat and/or a low fibre diet. In experimental and metabolic studies, both factors have been shown to influence bile secretion, colonic microflora, and bacterial enzymatic activity, thereby affecting concentration of faecal carcinogens (Wynder & Shigematsu, 1967; Hill, 1971; Burkitt, 1971; Reddy *et al.*, 1974). Beer consumption has also been implicated in the aetiology of colorectal cancer (Breslow & Enstrom, 1974; McMichael *et al.*, 1979). In addition, micronutrients have been suggested to affect colorectal carcinogenesis. High intakes of vitamin A (Phillips, 1975; Bjelke, 1980) and vitamin D/calcium (Garland *et al.*, 1985) have been associated with reduced risk of colorectal cancer while experimental studies have suggested that beta-carotene and vitamins C and E may be anti-carcinogens because of their antioxidant properties (Ames, 1983).

Major non-dietary aetiologic hypotheses include the role of exercise and, for women, the effect of reproductive factors. Vocational physical activity has been associated with reduced risk for colon cancer (Garabrant *et al.*, 1984; Vena *et al.*, 1985; Gerhardsson *et al.*, 1986). Physiologic events accompanying pregnancy, i.e., changes in hormonal profiles, may influence risk of colorectal cancer through a decrease in bile acid production (McMichael & Potter, 1980).

Despite reasonable biologic mechanisms for most of these hypotheses, epidemiologic studies have yielded conflicting results. The limitations of case-control studies in assessing dietary patterns and other lifestyle characteristics predating the disease may explain some of the observed inconsistencies.

The current study utilizes a prospective design to minimize bias due to the influence of disease on dietary and physical activity patterns and on recall of past diet and exercise. The study population is a predominantly white, upper-middle class retirement community where lifestyle habits tend to be stable and where losses to follow-up are minimal.

Correspondence: A.H. Wu.
Received 29 October 1986; and in revised form, 1 February 1987.

### Materials and methods

The study population consists of a large retirement community located about 50 miles south of Los Angeles, California. The residents of Leisure World, Laguna Hills, are almost entirely Caucasian and tend to be of the upper-middle socio-economic class. Women comprise about two-thirds of the population. A complete roster of community residents including address, date of birth, and date of move-in is routinely kept by the community business office and was made available to us for this survey.

Questionnaires were first mailed to all residents of the community on June 1, 1981. New residents who moved into the community between June, 1981 and June, 1982 were mailed this questionnaire on June 1982. After three mailings and follow-up telephone contact, 11,888 (62%) of the 19,152 residents returned the questionnaire.

The health survey included basic demographic information (birthdate, marital status, height, current weight, and weight at 21), medical history (including cancer and gallbladder surgery), personal habits (cigarette smoking, alcohol consumption, exercise), use of vitamin supplements and laxatives, and usual frequencies of consumption of 56 foods (or food groups) and coffee. Specific information on brand and formulation of vitamin supplements containing A, C, or E was obtained. Consumption of alcoholic beverages on an average weekday was asked separately for wine, beer and hard liquor and then combined to form an overall amount of alcohol consumed.* The amount of time per day spent in physical activities (e.g. swimming, biking, dancing) constituted the exercise variable.

On the basis of U.S. Department of Agriculture tables of food values (USDA; 1976–1984) for standard portion size (common household measure) of each item, we estimated average daily intake of vitamins A, C, D, beta-carotene and dietary fibre by summing the product of the respective nutrient content of each food item and its frequency of consumption. All 56 food items (vegetables, fruits, dairy products, liver, and cereal) contributed to the estimation of vitamin A intake. For the calculation of beta-carotene, vitamin C, dietary fibre, and dietary calcium, only the food items of relevance for the estimation of the particular

*The following assumptions and conversion rates were used: 1 oz. is equivalent to 30 ml; 1 glass of wine contains 120 ml, 1 bottle of beer, 360 ml, and 1 drink of hard liquor, 45 ml. The amount of absolute alcohol consumed per day is calculated using conversion of 10% for wine, 3.7% for beer, and 38% for hard liquor (Adams, 1975).

nutrient of interest were included.† When values for dietary fibre were not available, those for crude fibre were used. The food frequency method used in this mailed survey has been validated in a previous study which found that classification of individuals into tertiles by intakes based on the frequency method was both valid and comparable to classifications based on other dietary methods (index or regression) when intakes calculated by the in-person diet history method was the standard for comparison (Gray *et al.*, 1984). Each nutrient index was divided into tertiles on the basis of the distributions for all subjects in the cohort.

Pathological diagnosis of cancer among the cohort members are obtained from five local hospitals. At the time of the initial questionnaire, 85% of the study participants indicated they would receive medical care at one of these hospitals. Decedents are primarily identified from the files of the Orange County Health Department, but are supplemented when necessary by the community business office, the obituary columns in the neighbourhood newspapers, and relatives and friends. In addition, all participants are sent a follow-up questionnaire on a biennial basis. At the time of the last mailing beginning October 1985, letters to 17 individuals were returned as undeliverable. All other study participants returned the follow-up form, were spoken to on the phone, or were deceased, except for 5% for whom a current address but no phone number was available. All residents were followed until the diagnosis of colorectal cancer, death, or March 31, 1985 whichever occurred first.

To adjust for age, we divided the cohort into 4 age strata: $\leqq 64$, 65–74, 75–84, $\geqq 85$. Age-adjusted incidence rates were computed by direct standardization using the person-year distribution of the entire cohort as an internal standard (Lilienfeld *et al.*, 1967). Relative risks and *P*-values were obtained using a regression method that assumes that the occurrence of disease could be regarded as a Poisson process (implicit in the calculation of person-years at risk) with a constant hazard rate for a given person (Breslow *et al.*, 1983). The GLIM statistical software package program was used to make these calculations (Baker & Nelder, 1978). All reported *P*-values are two-sided. The 224 members of the cohort who reported pre-existing colorectal cancer were excluded from the at-risk population, leaving a cohort of 11,644 residents available for analysis.

Results are presented for colon and rectal cancer combined. However, analyses excluding rectal cancers ($n = 20$) did not substantially alter the results. The anatomic site was based on pathology reports, and we classified tumours from the caecum to splenix flexure as right-sided tumours and those from the descending colon to rectum as left-sided tumours.

†Foods included in food frequency questionnaire

*Vegetables:* asparagus, broccoli, brussel sprouts, cabbage, carrots, cauliflower, corn, green beans, green peas, green peppers (sweet), leafy greens, iceberg lettuce, other leafy lettuce, lima beans, red peppers (sweet), red peppers (hot), potatoes or turnips, summer squash, sweet potatoes, tomatoes, winter squash.

*Fruits:* apples, apricots, avocados, bananas, berries such as blackberries, cantaloupes, cherries, fruit cocktail, grapes, grapefruit and juice, honeydew and casaba melons, oranges and juice, papayas, peaches, persimmons, pineapple and juice, plums, prunes and juice, rhubarb, strawberries, watermelon.

*Other:* beef (calf) liver, pork liver, chicken (turkey) liver, eggs, super fortified cold cereals, other cold cereals, cooked cereals, whole grain bread, milk, cream, yogurt, cheese, butter and margarine, ice cream.

All foods in the list are included for the estimation of vitamin A, but the 'other' foods were excluded for the calculation of beta-carotene. The estimation of vitamin C was based on intake of all fruits, vegetables, and cold cereal. All fruits, vegetables, cereals and breads were included for the estimation of dietary fibre whereas dietary calcium was based on intake of dairy products. On the basis of data from the National Health and Nutrition Examination Survey (NHANES II) (Block *et al.*, 1985), the foods (or food groups) included in our questionnaire covered about 93%, 100%, and 85% of vitamin A, beta-carotene, and vitamin C respectively in the US diet. Values for dietary fibre were not available in the NHANES II survey. Compared to the Upstate New York Diet Study (Byers *et al.*, 1985), our assessment of dietary fibre covers about 89% of the foods contributing to fibre intake.

### Results

Table I presents the age-adjusted relative risks (RRs) and 95% confidence intervals (CIs) of colorectal cancer by sex for several health habits (alcohol use, smoking, physical activity, and coffee consumption), Quetelet's index (QI), use of laxatives, and history of gallbladder surgery. Risk of colorectal cancer was strongly associated with alcohol intake. Fifty-eight percent (M:71%, F:48%) of those who developed colorectal cancer drank some type of alcohol-containing beverage daily compared to only 45% (M:51%, F:41%) of the remainder of the cohort. The risks increased with increasing consumption of ethanol for both sexes combined. The RR was 1.5 (95% CI = 1.0, 2.4) among those who drank 1–30 ml (1 oz) per day compared to non-daily alcohol drinkers, and 1.9 (95% CI = 1.3, 2.9) among those who drank >30 ml per day relative to the same group. This effect of alcohol was highly significant in males ($P = 0.004$) but not in females ($P = 0.23$). The increased risk was due mainly to intake of hard liquor which accounted for about 70% of the ethanol consumed.

Quetelet's index was also positively related to risk of colorectal cancer. On the basis of the weight at entry, males in the middle and upper tertiles of QI experienced more than a two-fold increased risk compared to those in the lowest tertile ($P = 0.02$) although the risk was not highest for those in the highest tertile. In females, any affect of body mass was observed only for QI at age 21. Women in the highest tertile of QI at age 21 had 1.8 (95% CI = 1.0, 3.2) times the risk of those in the lowest tertile. Quetelet index at age 21 was not related to colorectal cancer risk in men.

Decreasing risks of colorectal cancer were observed with increasing levels of physical activity, especially in men, for whom results achieved statistical significance (*P*-value for trend = 0.008).

Smokers had higher risks of colorectal cancer than non-smokers but not as high as ex-smokers. The association with smoking was significant only for males ($P = 0.05$). No strong or significant associations were found between risk of colorectal cancer and coffee consumption, use of laxatives, or history of gallbladder surgery. The risk of colorectal cancer was actually lower among those who had had gall-bladder surgery.

Since alcohol, smoking, QI, and physical activity are intercorrelated, the independent effect of each was evaluated by inclusion of all these factors simultaneously in logistic regression models. In the model, alcohol and QI were defined as dichotomous variables whereas physical activity and smoking were coded in 3 dose levels (Table II, footnote). For males, alcohol, physical activity, QI and smoking each had a significant and independent effect on colorectal cancer risk and the variables entered the logistic regression model in this order. The adjusted RRs and 95% CI are presented on Table II. None of these four factors had a significant influence on colorectal cancer risk in females.

Table III presents age-adjusted relative risks of colorectal cancer in women for factors related to reproduction and hormone use. There were no significant associations between colorectal cancer and menarche, parity, age at first birth, or hormone use. Nulliparous women did not show the highest risk of colorectal cancer, but significantly decreasing risks were observed with increasing parity so that the risk of colorectal cancer among women with 3 or more children was about one-half that of women without children. Data on number of children were also available for men in the cohort. The RRs of colorectal cancer for men who had 1, 2,

**Table I** Age-adjusted relative risks and 95% confidence intervals (in parentheses) of colorectal cancer by selected factors

| | Total person-years[a] | No. of subjects | Relative risk and 95% CI Male | | Female | |
|---|---|---|---|---|---|---|
| Alcohol (ethanol) | | | | | | |
| Non-daily | 22,868 | 6,421 | 1.00 | | 1.00 | |
| 1–30 ml day$^{-1}$ | 9,131 | 2,537 | 2.24 | (1.1, 4.4) | 1.13 | (0.6, 2.1) |
| ≧31 ml day$^{-1}$ | 9,489 | 2,661 | 2.42 | (1.3, 4.5) | 1.45 | (0.8, 2.6) |
| Quetelet's index[b] | | | | | | |
| lower third | 11,911 | 3,429 | 1.00 | | 1.00 | |
| middle third | 14,752 | 4,091 | 2.80 | (1.3, 6.2) | 0.95 | (0.5, 1.8) |
| upper third | 14,640 | 4,042 | 2.40 | (1.1, 5.4) | 1.19 | (0.7, 2.2) |
| Physical activity | | | | | | |
| <1 hr day$^{-1}$ | 14,216 | 4,112 | 1.00 | | 1.00 | |
| 1–2 hr day$^{-1}$ | 14,377 | 3,979 | 0.89 | (0.5, 1.6) | 0.72 | (0.4, 1.3) |
| >2 hr day$^{-1}$ | 12,747 | 3,487 | 0.40 | (0.2, 0.8) | 0.89 | (0.5, 1.6) |
| Smoking | | | | | | |
| never | 20,129 | 5,593 | 1.00 | | 1.00 | |
| ex- >20 yrs[c] | 7,749 | 2,198 | 1.71 | (0.8, 3.6) | 1.61 | (0.8, 3.0) |
| ex- ≦20 yrs | 8,809 | 2,477 | 2.63 | (1.3, 5.3) | 0.71 | (0.3, 1.5) |
| current | 4,583 | 1,291 | 1.80 | (0.6, 5.2) | 1.35 | (0.7, 1.0) |
| Coffee | | | | | | |
| 0–1 cups day$^{-1}$ | 11,406 | 3,228 | 1.00 | | 1.00 | |
| 2–3 cups day$^{-1}$ | 25,646 | 7,161 | 1.32 | (0.7, 2.5) | 1.51 | (0.8, 2.7) |
| ≧4 cups day$^{-1}$ | 4,472 | 1,243 | 1.54 | (0.6, 3.7) | 1.17 | (0.4, 3.1) |
| Laxatives | | | | | | |
| <weekly | 33,749 | 9,229 | 1.00 | | 1.00 | |
| weekly | 2,549 | 738 | 1.45 | (0.5, 4.1) | 1.37 | (0.6, 3.2) |
| daily | 5,415 | 1,546 | 1.32 | (0.6, 2.7) | 1.38 | (0.7, 2.6) |
| Gallbladder surgery | | | | | | |
| no | 36,511 | 10,212 | 1.00 | | 1.00 | |
| yes | 47,91 | 1,356 | 0.51 | (0.2, 1.6) | 0.67 | (0.3, 1.6) |

[a]Total person-years do not always sum up to 41,531 due to persons with missing values for some variables. [b]Quetelet's index (weight height$^{-2}$ in lb in$^{-2}$) was divided in tertiles on the basis of the distribution for all subjects without colorectal cancer. The Quetelet's index for lower, middle, and upper tertile were respectively ≦31, 32–34, ≧35 for males and ≦29, 30–33, ≧34 for females. [c]Refers to the number of years since the respondent had stopped smoking.

**Table II** Effects of alcohol, physical activity, Quetelet's index and smoking on colorectal cancer risk in males according to logistic regression analysis

| Parameter | Estimate | Adjusted RR | 95% Confidence interval |
|---|---|---|---|
| Alcohol[a] | 0.7640 | 2.15 | 1.21–3.81 |
| Physical activity[b] | −0.4728 | 0.62 | 0.45–0.87 |
| Quetelet's index[c] | 0.9741 | 2.65 | 1.25–5.60 |
| Smoking[d] | 0.4007 | 1.49 | 1.06–2.09 |

[a]Daily drinkers compared with non-daily drinkers. [b]3 coded levels of physical activity (<1, 1–2, >2 h day$^{-1}$) were tested for a trend in risk with increasing activity in the model. [c]Upper two tertiles compared with lowest tertile of Quetelet's index. [d]3 coded levels of smoking (nonsmoker, ex-smoker who had stopped >20 yrs, and ex-smokers who had stopped ≦20 yrs plus current smokers) were tested for a trend in risk with increasing dose in the model. Physical activity and cigarette smoking were each treated as single coded variables with 3 dose levels since the trends for the 2 variables were statistically significant and the observed departure from trend were not statistically significant.

and 3 or more children compared to men with no children were 1.4 (95% CI = 0.7, 2.7), 0.8 (95% CI = 0.4, 1.6), and 0.4 (95% = 0.1, 1.1) respectively. For all types of menopause, risk increased with decreasing age at menopause, but the result was not statistically significant. Women who stopped menstruating as a result of hysterectomy (with or without removal of ovaries) had a RR of 1.8 (95% CI = 1.1, 2.9) compared to those who stopped menstruating naturally. The risk associated with surgical menopause was 1.7 (95% CI = 1.0, 2.9) after adjusting for age at menopause ($\chi^2_1 \leqq 3.42$, *P* value = 0.06).

Relative risks of colorectal cancer by intake of vitamins A and C, beta-carotene, dietary fibre, and calcium are shown in Table IV. Only dietary vitamin C was significantly associated with risk of colorectal cancer in females (*P* value for trend = 0.02); females with the lowest levels of consumption experienced about a two-fold increase in risk compared to those with the highest level of consumption. This apparent effect in females was diminished when rectal cancers ($n = 10$) were excluded in the analysis (*P* value for trend = 0.13). Neither vitamin C from supplements (Table IV) nor total vitamin C intake accounting for both dietary sources and supplements were related to colorectal cancer in either sex. Vitamins A and E from vitamin supplements (Table IV), and total vitamin A were also unrelated to risk of colorectal cancer.

In males, the influence of alcohol, physical activity, and QI were generally similar for right and left-sided cancers, although numbers became small in some categories. In females, any influence of alcohol, physical activity, and QI was limited to the left colon. The direction of the associations for left colon in females were similar to those observed in males, but none of these results were statistically significant (Table V).

**Table III** Age-adjusted relative risks and 95% confidence intervals (in parentheses) of colorectal cancer by selected reproductive factors

| | *Person-year*[a] | *No. of subjects* | *Relative risks and 95% CI* | |
|---|---|---|---|---|
| Menarche | | | | |
| ≦12 yr | 9,246 | 2,536 | 1.00 | |
| 13 yr | 8,330 | 2,296 | 0.83 | (0.5, 1.5) |
| ≧14 yr | 9,244 | 2,546 | 0.89 | (0.5, 1.6) |
| Every pregnant | | | | |
| yes | 19,611 | 5,306 | 1.00 | |
| no | 7,406 | 2,039 | 0.87 | (0.5, 1.5) |
| Number of children | | | | |
| 0 | 8,786 | 2,422 | 1.00 | |
| 1 | 5,606 | 1,554 | 1.56 | (0.9, 2.9) |
| 2 | 8,066 | 2,213 | 1.11 | (0.6, 2.0) |
| 3+ | 4,362 | 1,205 | 0.50 | (0.2, 1.3) |
| Age at first birth | | | | |
| <25 yr | 7,644 | 2,108 | 1.00 | |
| 25–29 yr | 6,783 | 1,874 | 0.80 | (0.4, 1.6) |
| ≧30 yr | 3,525 | 965 | 0.99 | (0.5, 2.1) |
| Type of menopause | | | | |
| natural | 18,380 | 5,056 | 1.00 | |
| hysterectomy, without bilateral oophorectomy | 3,985 | 1,090 | 1.16 | (0.6, 2.4) |
| bilateral oophorectomy, with or without hysterectomy | 2,434 | 668 | 2.16 | (1.1, 4.4) |
| hysterectomy, ovarian status unknown | 2,012 | 561 | 2.78 | (1.4, 5.5) |
| Age of menopause[b] | | | | |
| <45 yr | 7,148 | 1,974 | 1.00 | |
| 45–54 yr | 16,216 | 4,448 | 0.75 | (0.5, 1.3) |
| ≧55 yrs | 3,031 | 833 | 0.41 | (0.1, 1.2) |
| Years of oestrogens[c] | | | | |
| never | 11,701 | 3,252 | 1.00 | |
| <8 yr | 7,333 | 2,017 | 0.98 | (0.5, 1.8) |
| ≧8 yr | 7,539 | 2,043 | 1.02 | (0.6, 1.8) |

[a]Total person-years do not always sum add up to 27,017 due to persons with missing values for some variables or exclusions because the analysis was not applicable. [b]Among those who had natural menopause, the RRs were 1.00, 0.94 (95% CI = 0.5, 1.7), and 0.54 (95% CI = 0.2, 1.6) respectively. [c]Among those who had natural menopause, the RRs were 1.00, 0.93 (95% CI = 0.5, 1.7) and 0.80 (95% CI = 0.4, 1.5) respectively.

### Discussion

This study finds a significant relationship between alcohol intake, physical inactivity, and QI, and incidence of colorectal cancer in males. Although none of these associations were statistically significant in women, all, and especially alcohol consumption, were in the same direction.

Because of the short follow-up, we cannot rule out the possibility that early symptoms of colorectal cancer influenced responses to some of the study questions, such as dietary and physical activity patterns. To totally explore this possibility will require a longer period of follow-up.

Daily alcohol intake was associated with a significantly increased risk of colorectal cancer in this population. The evidence for a role of alcohol in colon carcinogenesis from previous studies is conflicting. International correlational studies have found a strong correlation between beer consumption, and especially, rectal cancer incidence, but, at best, only a weak association with wine and hard liquor consumption (Breslow & Enstrom, 1974). Previous case-control studies offer some further support. Potter and McMichael (1986) recently reported a positive association between colon and rectal cancers and total alcohol intake. In their study, unlike ours, results in women but not men were statistically significant. Like our study, spirits consumption rather than beer was more consistently associated with an increased risk. Williams and Horm (1977) also found a significant association between wine, beer, and hard liquor, individually, as well as for total alcohol intake, and colon cancer risk in men, but no association was found for rectal cancer. Paradoxically, in the same study, total alcohol intake was a significant risk factor for rectal but not colon cancer in women. In another study, a significantly increased risk for colorectal cancer with increased beer consumption was observed, but with only one of several comparison groups (Wynder & Shigematsu, 1967). In a fourth study, there was no evidence for an effect of alcohol overall in either sex, or of beer in males, but a significantly increased risk of rectal cancer with beer consumption was found in females (Miller *et al.*, 1983). Five studies found no statistically significant positive relationship between alcohol intake and colorectal cancer (Wynder *et al.*, 1969; Graham *et al.*, 1978; Dales *et al.*, 1979; Martinez *et al.*, 1979; Manousos *et al.*, 1983). Modan and coworkers (1975) reported a significant difference between cases and controls in alcohol intake but the direction of the association was not specified.

Results from cohort studies have been equally mixed. A dose-response relationship between risk of colorectal cancer and consumption of beer and liquor was reported in a cohort of Norwegian men (Bjelke, 1973). In a cohort study in Japan, daily beer drinkers experienced about a two-fold increase in risk for colon cancer (Hirayama, 1977), and there was a positive combined effect of alcohol and smoking in a later report in the same cohort (Hirayama, 1981). A cohort study of Japanese in Hawaii revealed a positive association between consumption of alcohol, primarily beer consumption, and rectal, but not colon cancer (Pollack *et al.*, 1985). A cohort study in Caucasian men employed at Western Electric Company found no significant relationship between ethanol intake and risk of colorectal cancer (Garland *et al.*, 1985).

There are no obvious explanations for the conflicting observations in these previous studies. Quality of data on alcohol use undoubtedly contributed to some of the discrepancies. Most of the case-control studies were dietary studies for which alcohol was not a major focus. Only one study developed a cumulative index of alcohol use, accounting for types, amounts, and duration of use (Williams & Horm, 1977), and like ours, found a strong association between total alcohol intake and risk of colon cancer in men. Moreover, all but one of the previous case-control studies (Potter & McMichael, 1986) were hospital-based studies. The failure to detect an association may be attributed in part to the use of hospital controls whose diagnoses may include alcohol-related diseases.

The mechanism by which alcohol entails an increased risk for colorectal cancer is not clear. One hypothesis for alcohol involves its effects on lipid secretion and metabolism. Bile cholesterol saturation is a function of the ratio of the concentrations of cholesterol to bile acids and phospholipids. Moderate alcohol intake decreases cholesterol saturation of bile by increasing bile acid concentration (Thorton *et al.*, 1983). Bile acid concentrations, in turn, may play a role in the formation and metabolism of faecal carcinogens.

Alcohol use is associated with a whole series of socio-cultural factors. While the association between alcohol use and colorectal cancer in our study was independent of some of these factors, such as smoking, weight, physical activity and various dietary factors, it is possible that an unidentified third variable, closely related to both alcohol use and colorectal cancer risk, may explain all or part of the observed association.

Our finding of a negative association between physical activity and colorectal cancer corroborate other recent studies indicating a protective role for physical activity, especially in men (Garabrant *et al.*, 1984; Vena *et al.*, 1985; Gerhardsson *et al.*, 1986). Previous work has been related mainly to occupational physical activity. Our study extends this observation to avocational activity. The stronger association in males than females observed in our study may be due, in part, to our index of physical activity, which did not include housework. Therefore our index may have

**Table IV** Age-adjusted relative risks and 95% confidence intervals (in parentheses) of colorectal cancer by dietary intake of selected nutrients and vitamin supplements

| | *Total person-years*[a] | *No. of subjects* | *Relative risks and 95% CI* | | | |
|---|---|---|---|---|---|---|
| | | | *Male* | | *Female* | |
| Vitamin A | | | | | | |
| Tertile | | | | | | |
| 1 (low) | 13,771 | 3,873 | 1.00 | | 1.00 | |
| 2 | 13,781 | 3,851 | 0.88 | (0.5, 1.7) | 1.01 | (0.6, 1.8) |
| 3 (high) | 13,766 | 3,840 | 1.08 | (0.6, 2.0) | 1.00 | (0.6, 1.8) |
| Beta-carotene | | | | | | |
| Tertile | | | | | | |
| 1 (low) | 13,745 | 3,864 | 1.00 | | 1.00 | |
| 2 | 13,716 | 3,839 | 0.89 | (0.5, 1.6) | 1.57 | (0.9, 2.9) |
| 3 (high) | 13,857 | 3,861 | 0.69 | (0.4, 1.3) | 1.20 | (0.6, 2.2) |
| Vitamin C | | | | | | |
| Tertile | | | | | | |
| 1 (low) | 14,110 | 4,001 | 1.00 | | 1.00 | |
| 2 | 14,263 | 3,966 | 1.01 | (0.6, 1.8) | 0.67 | (0.4, 1.2) |
| 3 (high) | 12,933 | 3,593 | 0.88 | (0.5, 1.7) | 0.50 | (0.3, 0.9) |
| Dietary Fibre | | | | | | |
| Tertile | | | | | | |
| 1 (low) | 14,589 | 4,110 | 1.00 | | 1.00 | |
| 2 | 14,843 | 4,137 | 0.60 | (0.3, 1.2) | 0.66 | (0.4, 1.2) |
| 3 (high) | 11,886 | 3,317 | 1.13 | (0.6, 2.1) | 0.64 | (0.4, 1.2) |
| Calcium | | | | | | |
| Tertile | | | | | | |
| 1 (low) | 13,875 | 3,865 | 1.00 | | 1.00 | |
| 2 | 13,840 | 3,873 | 1.19 | (0.6, 2.2) | 0.90 | (0.5, 1.6) |
| 3 (high) | 13,611 | 3,828 | 0.86 | (0.4, 1.7) | 0.89 | (0.5, 1.6) |
| Vitamin supplement A | | | | | | |
| none | 23,001 | 6,473 | 1.00 | | 1.00 | |
| $\leq$6500 IU day$^{-1}$ | 7,155 | 1,980 | 1.40 | (0.7, 2.7) | 1.01 | (0.5, 2.0) |
| $\geq$6600 IU day$^{-1}$ | 1,1172 | 3,117 | 0.95 | (0.5, 1.8) | 0.98 | (0.6, 1.7) |
| Vitamin supplement C | | | | | | |
| none | 15,329 | 4,342 | 1.00 | | 1.00 | |
| $\leq$350 mg day$^{-1}$ | 15,304 | 4,266 | 1.14 | (0.6, 2.1) | 1.04 | (0.6, 1.8) |
| $\geq$360 mg day$^{-1}$ | 10,678 | 2,956 | 1.11 | (0.6, 2.2) | 0.82 | (0.4, 1.6) |
| Vitamin supplement E | | | | | | |
| none | 19,452 | 5,499 | 1.00 | | 1.00 | |
| $\leq$350 mg day$^{-1}$ | 11,357 | 3,170 | 1.04 | (0.6, 1.9) | 0.92 | (0.5, 1.6) |
| $\geq$360 mg day$^{-1}$ | 10,526 | 2,902 | 0.83 | (0.4, 1.6) | 0.80 | (0.4, 1.5) |

[a]Total person-years are not the same due to persons with missing values for some variables.

substantially underestimated physical activity in some women.

The stronger association between QI and colorectal cancer in males compared to females in this study has also been described in other studies, although no biologic explanation has been offered (Lew & Garfinkel, 1979; Phillips & Snowdon, 1985). Other prospective studies, limited to men, have also found a positive association between weight and colorectal cancer risk (Garland *et al.*, 1985; Nomura *et al.*, 1985).

Obesity and low physical activity appeared to be independent risk factors, but they are undoubtedly correlated to some degree, and may have a similar underlying mechanism of action in colon carcinogenesis. Physical activity appears to stimulate colon peristalsis and to decrease random, nonpropulsive segmentation activity (Holdstock *et al.*, 1970). Potential carcinogens in faecal material are likely to have less contact with local colonic mucosa in persons who are highly active because of the decrease in mixing that occurs with segmentation and because of the shortened transit time of the stool. Our questionnaire focussed on defining vigorous *versus* sedentary lifestyle and may have been more appropriate for detecting these differences in men than in women; we did not measure the hours of active movement associated with housekeeping activities, which are likely to be associated preferentially with women in this population. More concise measures of the types and degree of physical activity inversely associated with colon cancer risk are needed and the relationship of physical activity to weight and weight gain requires further exploration. Although data on the relationship between physical activity and child rearing are not available, the similar protective effect of number of children in men and women in this population indicate that any parity effect in women may be a surrogate of other lifestyle factors.

In this cohort, increasing number of children was associated with substantial reduction in risk of developing colorectal cancer in both males and females, but in neither sex was the risk highest among those with no children. The role of parity in the aetiology of colorectal cancer is not clear. Three studies have found a significantly decreased risk associated with increasing parity in women (Weiss *et al.*, 1981; Potter & McMichael, 1983; McMichael & Potter, 1984); two of these studies also had pertinent data in men. In the first study, no data were available on number of children, but men with children had no reduction in risk compared to men with no children (Potter & McMichael, 1983). In the second study by the same investigators, the reduced risk with increasing number of children was limited to women (McMichael & Potter, 1984). The protective effect of increasing parity in females was not confirmed in 3 other studies (Byers *et al.*, 1982; Papadimitriou *et al.*, 1984; Howe

**Table V** Age-adjusted relative risks and 95% confidence intervals (in parentheses) of colorectal cancer by anatomic site

| *Males* | *Person-years* | *No. of subjects* | *Anatomic site*[a] *Right colon* | | *Left colon* | |
|---|---|---|---|---|---|---|
| Alcohol (ethanol) | | | | | | |
| non-daily | 7,074 | 2,039 | 1.00 | | 1.00 | |
| 1–30 ml day$^{-1}$ | 3,163 | 913 | 1.49 | (0.5, 4.2) | 3.18 | (1.2, 8.4) |
| ≧31 ml day$^{-1}$ | 4,199 | 1,211 | 2.84 | (1.2, 6.5) | 2.21 | (0.8, 6.0) |
| Physical activity | | | | | | |
| <1 h day$^{-1}$ | 3,655 | 1,132 | 1.00 | | 1.00 | |
| 1–2 h day$^{-1}$ | 4,796 | 1,374 | 0.93 | (0.4, 2.1) | 1.05 | (0.4, 2.3) |
| >2 h day$^{-1}$ | 5,962 | 1,649 | 0.50 | (0.2, 1.3) | 0.36 | (0.1, 1.1) |
| Quetelet's index | | | | | | |
| lower third | 4,076 | 1,231 | 1.00 | | 1.00 | |
| middle third | 4,771 | 1,354 | 2.88 | (0.9, 8.9) | 2.48 | (0.8, 7.8) |
| upper third | 5,525 | 1,556 | 2.36 | (0.8, 7.4) | 2.23 | (0.7, 7.1) |
| Females | | | | | | |
| Alcohol (ethanol) | | | | | | |
| non-daily | 15,794 | 4,382 | 1.00 | | 1.00 | |
| 1–30 ml day$^{-1}$ | 5,968 | 1,624 | 0.35 | (0.1, 1.5) | 1.70 | (0.9, 3.9) |
| ≧31 ml day$^{-1}$ | 5,290 | 1,450 | 1.00 | (0.4, 2.8) | 1.66 | (0.8, 3.6) |
| Physical activity | | | | | | |
| <1 h/day$^{-1}$ | 10,561 | 2,890 | 1.00 | | 1.00 | |
| 1–2 h day$^{-1}$ | 9,581 | 2,605 | 1.03 | (0.4, 2.7) | 0.57 | (0.3, 1.2) |
| >2 h day$^{-1}$ | 6,785 | 1,838 | 1.16 | (0.4, 2.5) | 0.68 | (0.3, 1.5) |
| Quetelet's index | | | | | | |
| lower third | 7,835 | 2,198 | 1.00 | | 1.00 | |
| middle third | 9,981 | 2,737 | 1.08 | (0.4, 3.1) | 0.92 | (0.4, 2.1) |
| upper third | 9,115 | 2,486 | 1.35 | (0.5, 3.8) | 1.33 | (0.6, 2.9) |

[a]Right colon tumours included those located in the caecum to splenix flexure, and left colon tumours included those located in the descending colon to rectum. Two male and 4 female colon cancers were classified as colon cancer NOS on the pathology report and they were excluded in the analysis.

*et al.*, 1985), two of which (Byers *et al.*, 1982; Papadimitriou *et al.*, 1984) actually showed non-significant increasing risks with increasing parity. The lowered HDL-cholesterol level and the decreased bile acid production associated with parity have been proposed to explain the purported protective effect of parity (McMichael & Potter, 1980). The conflicting evidence from previous studies combined with our observation of a similar protective effect in men suggests a non-causal relationship resulting from other specific life-style variations such as physical activity associated with large families.

The significance of our finding of an increased risk associated with artificial menopause is unclear and also has little support from previous studies. Weiss *et al.* (1981) found no association between hysterectomy status and risk of colorectal cancer but it is not clear if hysterectomy after menopause was included in their analysis. The lack of an association with hormone use in our study is compatible with results from other investigators (Weiss *et al.*, 1981; Potter & McMichael, 1983).

In this population, there was no evidence that dietary vitamin A or E, beta-carotene, calcium, or dietary fibre provide any significant protection against colorectal cancer. However, it must be emphasized that our cohort members are predominantly of the upper-middle class and apparently well-fed with less than 5 percent receiving less than the 1980 recommended dietary allowance for vitamin A (Food & Nutrition Board, 1980). We observed a significant inverse association between dietary (but not supplemental or total) vitamin C and risk of colorectal cancer in females. The possible role of vitamin C in the aetiology of rectal cancer warrants further study since there is supportive evidence from a recent study in Australia (Potter & McMichael, 1986), although no protective effect was found for colon or rectal cancer in a Canadian study (Jain *et al.*, 1980).

Prospective serological studies have found no relation between plasma vitamin A or beta-carotene and colorectal cancer (Stahelin *et al.*, 1984; Nomura *et al.*, 1985) although plasma vitamin E and C levels were lower in colorectal cancer cases than in controls in one of these studies (Nomura *et al.*, 1985). Previous results on the role of dietary fibre have been equally mixed. Early correlational studies based on estimates of crude fibre intake revealed no association between fibre intake and colon cancer incidence (Drasar & Irving *et al.*, 1973; Liu *et al.*, 1979), whereas a study of Bingham and coworkers using estimates of dietary fibre suggested an inverse association between pentose fibre and colon cancer incidence (Bingham *et al.*, 1979). However, a more recent study using a refined measurement of dietary fibre did not confirm this earlier work (Bingham *et al.*, 1985). While two case-control studies offered supportive evidence (Modan *et al.*, 1975; Dales *et al.*, 1979), three others found no association between crude or dietary fibre and colorectal cancer risk (Jain *et al.*, 1980; Miller *et al.*, 1983; Potter & McMichael, 1986).

We found no association between vitamin supplements and colorectal cancer and are not aware of published data relating the effects of vitamins A, C, or E from vitamin supplements to colorectal cancer risk. If these exposures are unstable over time, as might occur if use of vitamins is a function of length of residence in the community, then it is possible that only effects that are important late in carcinogenesis will be detected in this study because of the short follow-up period. Fifty-six per cent of our cohort reported taking vitamin supplements for over 10 years and 36% for over 20 years, suggesting that for most residents, use of vitamin supplements was long standing. Unfortunately we have no data on duration of use of specific supplements.

Dietary fat, a major dietary hypothesis, was not addressed by our study. Although fat intake is unlikely to be strongly related to alcohol use (Bebb *et al.*, 1971; Jones *et al.*, 1982), it may confound the observed associations with physical activity and QI. However, fat has not been consistently associated with colorectal cancer risk in analytic epidemiologic studies (Dales *et al.*, 1979; Haenszel *et al.*, 1980; Stemmermann *et al.*, 1985) and, in some, has been unrelated to body weight (Bebb *et al.*, 1971; Jones *et al.*, 1982).

While major hypotheses regarding colon carcinogenesis have been proposed for dietary fibre and various micronutrients, with the notable exception of a possible effect of vitamin C in women, we were unable to confirm any of these hypotheses.

We thank Joan Howland and Janie Teran for preparation of the manuscript.

### References

ADAMS, D.F. (1975). *Nutritive value of American foods in common units.* USDA agriculture handbook No. 456, U.S. Government Printing Office: Washington D.C.

AMES, B.N. (1983). Dietary carcinogens and anticarcinogens. *Science,* **221,** 1256.

BAKER, R.J., NELDER, J.A. (1978). *The GLIM System: Release 3.* Numerical Algorithms Group: Oxford.

BEBB, H.T., HOUSER, H.B., WITSCHI, J.C., LITTELL, A.S., FULLER, R.K. (1971). Calorie and nutrient contribution of alcoholic beverages to the usual diets of 155 adults. *Am. J. Clin. Nutr.,* **24,** 1042.

BINGHAM, S., WILLIAMS, D.R.R., COLE, T.J. & JAMES, W.P.T. (1979). Dietary fibre and regional large-bowel cancer mortality in Britain. *Br. J. Cancer,* **40,** 456.

BINGHAM, S.A., WILLIAMS, D.R.R. & CUMMINGS, J.H. (1985). Dietary fibre consumption in Britain: New estimates and their relation to large bowel cancer mortality. *Br. J. Cancer,* **52,** 399.

BJELKE, E. (1973). *Epidemiologic studies of cancer of stomach, colon and rectum; with special emphasis on the role of diet.* Ph.D. Thesis. University of Minnesota, Vol. I–IV. Ann Arbor.

BJELKE, E. (1980). Epidemiology of colorectal cancer, with emphasis on diet. In *Human Cancer: its characteristics and treatment,* Davis W. (ed) p. 158. Excerpta Medica: Amsterdam.

BLOCK, G., DRESSER, C.M., HARTMAN, A.M. & CARROLL, M.D. (1985). Nutrient sources in the American diet: Quantitative data from the NHANES II survey. *Am. J. Epidemiol.,* **122,** 13.

BYERS, T., GRAHAM, S. & SWANSON, M. (1982). Parity and colorectal cancer risk in women. *J. Natl Cancer Inst.,* **69,** 1059.

BYERS, T., MARSHALL, J., FIEDLER, R., ZIELEZNY, M. & GRAHAM, S. (1985). Assessing nutrient intake with an abbreviated dietary interview. *Am. J. Epidemiol.,* **122,** 41.

BRESLOW, N.E. & ENSTROM, J.E. (1974). Geographic correlation between cancer mortality rates and alcohol-tobacco consumption in the United States. *J. Natl Cancer Inst.,* **53,** 631.

BRESLOW, N.E., LUBIN, J.H., MAREK, P. LANGHOLZ, B. (1983). Multiplicative models and cohort analysis. *J. Amer. Surg. Assoc.,* **78,** 1.

BURKITT, D.P. (1971). Epidemiology of cancer of the colon and rectum. *Cancer,* **28,** 3.

DALES, L.G., FRIEDMAN, G.D., URY, H.K., GROSSMAN, S. & WILLIAMS, S.R. (1979). A case-control study of relationships of diet and other traits to colorectal cancer in American Blacks. *Am. J. Epidemiol.,* **109,** 132.

DRASAR, B.S. & IRVING, D. (1973). Environmental factors and cancer of the colon and breast. *Br. J. Cancer,* **27,** 167.

FOOD AND NUTRITION BOARD, NATIONAL RESEARCH COUNCIL. (1980). Recommended dietary allowance. 9th ed. National Academy Press: Washington, DC.

GARABRANT, D.H., PETERS, J.M., MACK, T.M. & BERNSTEIN, L. (1984). Job activity and colon cancer risk. *Am. J. Epidemiol.,* **119,** 1005.

GARLAND, C., SHEKELLE, R.B., BARRETT-CONNOR, E., CRIQUI, M.H., ROSSOF, A.H. & PAUL, O. (1985). Dietary vitamin D and calcium and risk of colorectal cancer: a 19-year prospective study in men. *Lancet,* **i,** 307.

GERHARDSSON, M., NORELL, S.E., KIVARANTA, H., PEDERSEN, N.L. & AHLBORN, A. (1986). Sedentary jobs and colon cancer. *Am. J. Epidemiol.,* **123,** 775.

GRAHAM, S., DAYAL, H., SWANSON, M., MITTELMAN, A. & WILKINSON, G. (1978). Diet in the epidemiology of cancer of the colon and rectum. *J. Natl Cancer Inst.,* **61,** 709.

GRAY, G.E., PAGANINI-HILL, A., ROSS, R.K. & HENDERSON, B.E. (1984). Assessment of three brief methods of estimation of vitamin A and C intakes for a prospective study of cancer: Comparison with dietary history. *Am. J. Epidemiol.,* **119,** 581.

HAENSZEL, W. & KURIHARA, M. (1968). Studies of Japanese migrants. I. Mortality from cancer and other diseases among Japanese in the United States. *J. Natl Cancer Inst.,* **40,** 43.

HAENSZEL, W., LOCKE, F.B. & SEGI, M. (1980). A case-control study of large bowel cancer in Japan. *J. Natl. Cancer Inst.,* **64,** 17.

HILL, M.J. (1971). The effect of some factors on the faecal concentration of acid steroids, neutral steroids and urobilins. *J. Pathol.,* **104,** 239.

HIRAYAMA, T. (1977). Changing patterns of cancer in Japan with special reference to the decrease in stomach cancer mortality. In *Origins of Human Cancer. Cold Spring Harbor Conference on Cell Proliferation,* Hiatt, H.H., Watson, J.D. & Winsten, J.A. (eds) Book A, p. 55. Cold Spring Harbor Laboratory: New York.

HIRAYAMA, T. (1981). A large-scale cohort study on the relationship between diet and selected cancers of digestive organs. In *Banbury Report 7: Gastro-intestinal Cancer: Endogenous factors,* Bruce, W.R. & 2 others (eds) p. 409. Cold Spring Harbor Laboratory: New York.

HOLDSTOCK, D.J., MISIEWICZ, J.J. & SMITH, T. (1970). Propulsion (mass movements) in the human colon and its relationship to meals and somatic activity. *Gut,* **11,** 91.

HOWE, G.R., CRAIB, K.J.P. & MILLER, A.B. (1985). Age at first pregnancy and risk of colorectal cancer: A case-control study. *J. Natl Cancer Inst.,* **74,** 1155.

JAIN, M., COOK, G.M., DAVIS, F.G., GRACE, M.G., HOWE, G.R. & MILLER, A.B. (1980). A case-control study of diet and colo-rectal cancer. *Int. J. Cancer,* **26,** 757.

JONES, B.R., BARRETT-CONNOR, E., CRIQUI, M.H. & HOLDBROOK, M.J. (1982). A community study of calorie and nutrient intake in drinkers and nondrinkers of alcohol. *Am. J. Clin. Nutr.,* **35,** 135.

LEW, E.A. & GARFINKEL, L. (1979). Variations in mortality by weight among 750,000 men and women. *J. Chron Dis.,* **32,** 563.

LILIENFELD, A.M., PEDERSEN, E. & DOWD, J.E. (1967). *Cancer Epidemiology: Methods of Study.* Johns Hopkins Press: Baltimore.

LIU, K., STAMLER, J., MOSS, D., GARSIDE, D., PERSKY, V. & SOLTERO, I. (1979). Dietary cholesterol, fat and fibre and colon cancer mortality. *Lancet,* **ii,** 782.

McMICHAEL, A.J., POTTER, J.D. & HETZEL, B.S. (1979). Time trends in colorectal cancer mortality in relation to food and alcohol consumption: United States, United Kingdom, Australia and New Zealand. *Int. J. Epidemiol.,* **8,** 295.

McMICHAEL, A.J. & POTTER, J.D. (1980). Reproduction, endogenous and exogenous sex hormones and colon cancer: A review and hypothesis. *J. Natl Cancer Inst.,* **65,** 1201.

McMICHAEL, A.J. & POTTER, J.D. (1984). Parity and death from colon cancer in women: A case-control study. *Community Hlth Studies,* **8,** 19.

MANOUSOS, O., DAY, N.E., TRICHOPOULOS, D., GEROVASSILIS, F., TZONOU, A. & POLYCHRONOPOULOU, A. (1983). Diet and colorectal cancer: A case-control study in Greece. *Int. J. Cancer,* **32,** 1.

MARTINEZ, I., TORRES, R., FRIAS, Z., COLÓN, J.R. & FERNANDEZ, N. (1979). Factors associated with adenocarcinomas of the large bowel in Puerto Rico. *Adv. Med. Onc. Res. Educ.,* **3,** 45.

MILLER, A.B., HOWE, G.R., JAIN, M., CRAIB, K.J.P. & HARRISON, L. (1983). Food items and food groups as risk factors in a case-control study of diet and colorectal cancer. *Int. J. Cancer,* **32,** 155.

MODAN, B., BARRELL, V., LUBIN, F., MODAN, M., GREENBERG, R.A. & GRAHAM, S. (1975). Low-fiber intake as an etiologic factor in cancer of the colon. *J. Natl Cancer Inst.,* **55,** 15.

NOMURA, A., STEMMERMANN, G.N., HEILBRUN, L.K., SALKELD, R.M. & VUILLEUMIER, J.P. (1985). Serum vitamin levels and the risk of cancer of specific sites in men of Japanese ancestry in Hawaii. *Cancer Res.,* **45,** 2369.

NOMURA, A., HEILBRUN, L.K. & STEMMERMANN, G.N. (1985). Body mass index as a predictor of cancer in men. *J. Natl Cancer Inst.,* **74,** 319.

PAPADIMITRIOU, C., DAY, N. & TZONOU, A. (1984). Biosocial correlates of colorectal cancer in Greece. *Int. J. Epidemiol.*, **13**, 155.

PHILLIPS, R.L. (1975). Role of life-style and dietary habits in risk of cancer among Seventh-Day Adventists. *Cancer Res.*, **35**, 3513.

PHILLIPS, R.L. & SNOWDON, D.A. (1985). Dietary relationships with fatal colorectal cancer among Seventh-Day Adventists. *J. Natl Cancer Inst.*, **74**, 307.

POLLACK, E.S., NOMURA, A.M., HEILBRUN, L.K., STEMMERMANN, G.N. & GREEN, S.B. (1985). Prospective study of alcohol consumption of cancer. *N. Engl. J. Med.*, **310**, 617.

POTTER, J.D. & McMICHAEL, A.J. (1983). Large bowel cancer in women in relation to reproductive and hormonal factors: a case-control study. *J. Natl Cancer Inst.*, **71**, 703.

POTTER, J.D. & McMICHAEL, A.J. (1986). Diet and cancer of the colon and rectum: a case-control study. *J. Natl Cancer Inst.*, **76**, 557.

REDDY, B.S., WEISBURGER, J.H. & WYNDER, E.L. (1974). Faecal bacterial B-glucuronidase: Control by diet. *Science*, **183**, 416.

STAHELIN, H.B., ROSEL, F., BUESS, E. & BRUBACHER, G. (1984). Cancer, vitamins, and plasma lipids: Prospective Basel Study. *J. Natl Cancer Inst.*, **73**, 1463.

STEMMERMANN, G., NOMURA, A., HEILBRUN, L.K., MOWER, H. & HAYASHI, T. (1985). Colorectal cancer in Hawaiian Japanese men: a progress report. *Natl Cancer Inst. Monogr.*, **69**, 125.

THORTON, J., SYMES, C. & HEATON, K. (1983). Moderate alcohol intake reduces bile cholesterol saturation and raises HDL cholesterol. *Lancet*, **ii**, 819.

U.S. DEPARTMENT OF AGRICULTURE. (1976–1984). *Composition of Foods. Handbooks Nos. 8.1 to 8.14.* U.S. Government Printing Office: Washington D.C.

VENA, J.E., GRAHAM, S., ZIELZENY, M., SWANSON, M.K., BARNES, R.E. & NOLAN, J. (1985). Lifetime occupational exercise and colon cancer. *Am. J. Epidemiol.*, **122**, 257.

WATERHOUSE, J., MUIR, C., SHANMUGARATNAM, K. & POWELL, J. (eds) (1982). *Cancer Incidence in Five Continents.* **IV.** IARC Sci. Publ., No. 42, Lyon.

WEISS, N.S., DALING, J.R. & CHOW, W.H. (1981). Incidence of cancer of the large bowel in women in relation to reproductive and hormonal factors. *J. Natl Cancer Inst.*, **67**, 57.

WHITTEMORE, A.S., ZHENG, S., WU, A. & 7 others (1985). Colorectal cancer in Chinese and Chinese-Americans. *Natl Cancer Inst., Monogr.*, **69**, 43.

WILLIAMS, R.R. & HORM, J.W. (1977). Association of cancer sites with tobacco and alcohol consumption and socioeconomic status of patients: interview study from the Third National Cancer Survey. *J. Natl Cancer Inst.*, **58**, 325.

WYNDER, E.L. & SHIGEMATSU, T. (1967). Environmental factors of cancer of the colon and rectum. *Cancer*, **20**, 1520.

WYNDER, E.L., KAJITANI, T., ISHIKAWA, S., DODO, H. & TAKANO, A. (1969). Environmental factors of cancer of the colon and rectum. II. Japanese epidemiological data. *Cancer*, **23**, 1210.